# Board-invited review: Sensitivity and responses of functional groups to feed processing methods on a molecular basis

**DOI:** 10.1186/2049-1891-3-40

**Published:** 2012-12-10

**Authors:** Peiqiang Yu

**Affiliations:** 1 Department of Animal and Poultry Science, College of Agriculture and Bioresources, University of Saskatchewan, 51 Campus Drive, Saskatoon, S7N 5A8, Canada; 2 Professor and Ministry of Agriculture Strategic Research Chair, (教授,农业部战略研究主席), College of Agriculture and Bioresources, University of Saskatchewan, 51 Campus Drive, Saskatoon, S7N 5A8, Canada

**Keywords:** Sensitivity and responses of functional groups, Molecular structures, Feed processing, Nutrient utilization and availability

## Abstract

In complex feed structures, there exist main chemical functional groups which are associated with nutrient utilization and availability and functionality. Each functional group has unique molecular structure therefore produce unique molecular vibration spectral profile. Feed processing has been used to improve nutrient utilization for many years. However, to date, there was little study on processing-induced changes of feed intrinsic structure and functional groups on a molecular basis within intact tissue. This is because limited research technique is available to study inherent structure on a molecular basis. Recently bioanalytical techniques: such as Synchrotron Infrared Microspectroscopy as well as Diffuse Reflectance Infrared Fourier Transform molecular spectroscopy have been developed. These techniques enable to detect molecular structure change within intact tissues. These techniques can prevent destruction or alteration of the intrinsic protein structures during processing for analysis. However, these techniques have not been used in animal feed and nutrition research. The objective of this review was show that with the advanced technique, sensitivity and responses of functional groups to feed processing on a molecular basis could be detected in my research team. These functional groups are highly associated with nutrient utilization in animals.

## Introduction

### Functional groups in feeds

Each feed has a unique structure. The unique structure results in unique molecular vibrational spectra. Functional groups are associated within unique feed intrinsic structure. These functional groups consist of macromolecular such as lipid, protein, structural and non-structural carbohydrates which are nutrient for animal and human. Under infrared vibrational molecular spectroscopy, these functional groups produced unique bands [1]. From the IR spectrum (4000–500 cm^-1^) the presence or absence of various organic functional groups is readily observed [2-4].

### Molecular vibration of functional groups

Several relatively unique absorption of lipid vibration spectra (4000–500 cm^-1^) usually locate at several regions in mid-infrared regions. (a) It located at ca. 2800~3000 cm^-1^, asymmetric and symmetric stretching vibrations of CH_3_ (ca. 2956 and 2874 cm^-1^) and CH_2_ (ca. 2922 and 2852 cm^-1^) groups of acyl chains [5-8]. (b) it locates at ca. 3015 cm^-1^, unsaturation (CH attached to C=C). (c) It locate at ca. 1800–1500 cm^-1^ at ca. 1740 cm^-1^ stretching vibration of the carbonyl ester C=O bond [2,5]. The protein produces relative unique bands which are amide I and amide II at ca 1710–1480 cm^-1^. The amide I band arises predominantly from the C=O stretching vibration of the peptide bond and particularly sensitive to and can be used to predict protein secondary structure [5-8]. The relative unique absorption bands from carbohydrates are complex. Normally carbohydrate bands are found at the 1180–900 cm^-1^ region of the infrared spectrum. There are some unique carbohydrate bands, such as band at 1420 cm^-1^ for β-glucan [2], bands at ca. 1420, 1370 and 1335 cm^-1^ for structural carbohydrates [2,3], band at ca. 1025 cm^-1^ for starch in endosperm of cereal grain [2,3], band at ca. 1240 cm^-1^ for hemicellulose. These functional groups are highly related to nutrient availability [9,10]

### Molecular spectroscopy and spectral analysis

There are several molecular spectroscopy that could be used for molecular structure study for feeds. First one is cutting-edge bioanalytical technique: synchrotron-radiation based technique SR-IMS. This technique enables to detect molecular structure change within intact tissues within cellular and subcellular dimensions [11-14]. This technique can prevent destruction or alteration of the intrinsic protein structures during processing for analysis. Other molecular spectroscopy technique could be used for feed inherent structure study included ATR/FT/IR spectroscopy [15], DRIFT molecular spectroscopy [16]. For molecular spectral analysis, it includes univariate and multivariate analyses [4,17-19]. For univariate analysis, we could determine each functional group intensity and distribution within intact feeds tissue. For multivatiate analysis, there are two methods are commonly used in my research team to discriminate and classify feed molecular structures and processing-induced structure changes, such as heating, autoclaving, bioethnaol processing and gene transformation [20]. The first one is agglomerative hierarchical cluster analysis (AHCA). AHCA is initially used to try to determine the main sources of variation in SRFTIRM protein spectra and the results are displayed as dendrograms using Ward’s method (Cytospec Software).

### Review objective

However, these molecular-based techniques have not been used in animal feed and nutrition research. The objective of this review was to show that with the advanced technique, sensitivity and responses of functional groups to feed processing on a molecular basis could be detected in my research team.

## Application I

### Processing-induced changes in lipid-related function groups on a molecular basis

In one of our research [21], we studied heat-induced changes to lipid molecular structure in Vimy flaxseed: spectral intensity and molecular clustering. In this study, we characterized any changes of each functional groups mainly associated with lipid structure in flaxseed (*Linum usitatissimum*, cv. Vimy), that occurred on a molecular level during the treatment process using infrared molecular spectroscopy. We autoclaved flaxseed samples at 120°C for 20, 40 or 60 min for treatments 1, 2 and 3, respectively. The lipid CH_3_ asymmetric (ca. 2959 cm^-1^), CH_2_ asymmetric (ca. 2928 cm^-1^), CH_3_ symmetric (ca. 2871 cm^-1^) and CH_2_ symmetric (ca. 2954 cm^-1^) functional groups, lipid carbonyl C=O ester group (ca. 1745 cm^-1^), lipid unsaturation group (CH attached to C=C) (ca. 3010 cm^-1^) and their intensity ratios were determined. We also used hierarchical cluster analysis (CLA) and principal components analysis (PCA) to identify molecular structure difference. The results showed that there were linear and quadratic effects of the treatment time from 0, 20, 40 and 60 min on the ratios of the CH_2_ asymmetric to CH_3_ asymmetric stretching vibration intensity. But autoclaving had no significant effect on lipid carbonyl C=O ester group and lipid unsaturation group (CH attached to C=C). The results indicated that autoclaving had different impact on different functional groups which are related to lipid and molecular spectroscopy was able to pick up heat-induced changes in lipid conformation. A future study is needed to quantify the relationship between lipid molecular structure changes and functionality/availability [21]. In another study [22], we reported sensitivity of molecular functional groups to bioethanol processing in lipid biopolymer of co-products using DRIFT molecular spectroscopy.

## Application II

### Processing-induced changes in protein-related function groups on a molecular basis

The sensitivity and responses of protein-related functional group to various processing has been reviewed in details and published in Molecular Nutrition and Food [20]. In this article we have reported the effects of various processing included gene-transformation, bioethanol processing, autoclaving and dry roasting on protein molecular structure changes in feeds. We also reported the responses of each functional group to the processing. The results show, again, different functional groups have different sensitivity to different processing. In Yu et al. [23], study, we used advanced synchrotron technique- SR-IMS to compare protein molecular structure of alfalfa plant tissues transformed with the maize *Lc* regulatory gene with non-transgenic alfalfa protein within cellular and subcellular dimensions and to quantify protein inherent structure profiles using Gaussian and Lorentzian methods of multi-component peak modeling [17,24]. It was found that transgenic *Lc* alfalfa leaves contain similar proteins to non-transgenic alfalfa (because amide I and II intensities were identical), but a subtle differences in protein molecular structure. Further study is needed to understand the relationship between these structural profiles and biological features such as protein nutrient availability, protein bypass and digestive behavior of livestock fed with this type of forage [23]. In the study by Doiron et al. [25], it was found that autoclaving changed protein structure α- helix to β-sheet ratio and protein sub-fractions. The protein structure α-helix to β-sheet ratio had significantly positive correlation with total intestinally absorbed protein supply and negative correlation with degraded protein balance [25]. In the study bioethanol processing study, it was found that bioethanol processing changed protein molecular structure α-helix to β-sheet ratio and protein amide I to II ratio which highly related to nutrient values. The results indicated that the protein structure α-helix to β-sheet ratio in the new co-products of bioethanol productions are correlated to total intestinally absorbed protein supply.

## Application III

### Processing-induced changes in carbohydrate-related function groups on a molecular basis

Feed processing has different impact on different functional groups in feeds which are related to carbohydrate. Yu [26]**,** reported the study on effect of bioethanol processing on carbohydrate functional groups by using DRIFT molecular spectroscopy. The following functional groups were studied, including: “A_Cell (peaks area region and baseline: ca. 1485–1188 cm^-1^) mainly associated with hemi- and cellulosic compounds**,** A_1240 (peak area centered at ca.1240 cm^-1^ with region and baseline: ca. 1292–1198 cm^-1^) mainly associated with cellulosic compounds, A_CHO (peaks region and baseline: ca. 1187–950 cm^-1^) associate with total CHO, A_928 (peak area centered at ca. 928 cm^-1^ with region and baseline: ca. 952–910 cm^-1^) and A_860 (peak area centered at ca. 860 cm^-1^ with region and baseline: ca. 880–827 cm^-1^) mainly associated with non-structural CHO, H_1415 (peak height centered at ca.1415 cm^-1^ with baseline: ca. 1485–1188 cm^-1^) and H_1370 (peak height at ca.1370 cm^-1^ with a baseline: ca. 1485–1188 cm^-1^) mainly associated with structural CHO”. The authors found that A_Cell, A_CHO, H_1415 and H_1370 had no correlation with CHO chemical and nutrient profiles in the bioethanol co-products. However, CHO molecular spectral intensity of the A_1240, A_928 and A_860 had strong correlation with rapidly degradable CHO fraction (CB1), lowly degradable CHO fraction **(**CB2) and an unavailable CHO fraction (CC) and could be a good indicator. In conclusion, the changes of the CHO molecular structures during the processing for bioethanol production were highly associated with carbohydrate degradable subfractions in ruminants**.**

## Conclusions and implications

The review shows that molecular spectroscopy technique could be used to study the sensitivity and response of functional groups in complex feed systems to various feed processing on molecular basis. It has a potential to study processing impact directly on feed and nutrient utilization and availability. These functional groups are highly associated with nutrient utilization in animals.

## Competing interests

The authors declare that they have no competing interests.
